# The effect of fetal movement awareness on birth outcomes among Somali migrant women – findings from a non-randomised intervention study in Sweden

**DOI:** 10.1186/s12884-026-08707-z

**Published:** 2026-01-28

**Authors:** Anna Andrén, Ingela Rådestad, Helena Lindgren, Kerstin Erlandsson, Viktor Skokic, Anna Akselsson

**Affiliations:** 1 Department of Health Promoting Science, Sophiahemmet University, Box 5605, Stockholm, 114 86 Sweden; 2 Department of Women’s and Children’s Health, Karolinska Institutet, Stockholm, Sweden; 3 School of Health and Welfare, Dalarna University, Falun, Sweden; 4 Department of Pelvic Cancer, Department of Molecular Medicine and Surgery, Karolinska University Hospital, Karolinska Institutet, Stockholm, Sweden

**Keywords:** Decreased fetal movements, Mindfetalness, Apgar score, Newborn health, Stillbirth prevention, Migrant health, Complex interventions

## Abstract

**Background:**

Women born in Somalia who have migrated to Sweden have increased risks of complications related to pregnancy and childbirth. Swedish-Somali women are, compared to women born in Sweden, less likely to contact healthcare for decreased fetal movements, which can be a sign of fetal distress. This study aimed to evaluate the effect of a Mindfetalness-based intervention in increasing awareness of fetal movements and its impact on birth outcomes among Swedish-Somali migrant women.

**Methods:**

An intervention study with a non-randomised control group, encompassing Swedish-Somali migrant women, with a singleton pregnancy, giving birth from gestational week 32 + 0 between June 2022 and June 2024. Data was collected from the Swedish Pregnancy Register and the Swedish Neonatal Quality Register. The intervention group consisted of 1,251 women who, in addition to routine information about fetal movements, also received information about Mindfetalness, a concept supporting women to become familiar with the baby’s movement pattern. The control group consisted of 1,555 women who only received routine information about fetal movement. Outcome measures included Apgar score at five minutes after birth, birth weight, neonatal intensive care unit admission, fetal death, onset of labour, mode of birth, and gestational age at birth. Log-binomial regression analyses were applied to assess associations between the intervention and selected outcomes.

**Results:**

There was a statistically significant increase in spontaneous vaginal births in the intervention group (RR 1.06, CI 1.01–1.11). No statistically significant differences between the intervention and control groups were observed for any other outcomes. Nearly one-third of women in both groups experienced a prolonged or post-term birth, while two-thirds were overweight or obese. The overall stillbirth rate ranged between 0.3% and 0.4%.

**Conclusions:**

The Mindfetalness intervention was associated with an increased incidence of spontaneous vaginal births, but no effect on other birth outcomes was observed. Implementing health interventions in a high-quality healthcare setting present challenges. When designing interventions to improve birth outcomes, it is essential to consider demographic data and the prevalence of risk factors within the target population. Further research involving stakeholders representing Swedish-Somali women is needed to understand the implications of this intervention.

**Trial registration:**

Retrospectively registered at ClinicalTrials.gov: NCT05540639, 30/06/2022.

**Supplementary Information:**

The online version contains supplementary material available at 10.1186/s12884-026-08707-z.

## Introduction

Women who have migrated from sub-Saharan African to Sweden have increased risks of pregnancy and birth related complications that may endanger both their own and their newborns’ health [1]. While the incidence of stillbirth among the majority of the Swedish population has steadily decreased over the past decades, a recently published study conducted in Region Stockholm found that, on the contrary, it has increased for women born in sub-Saharan Africa [2]. These disparities are not unique to the Swedish context; they are also observed in other Nordic countries, as well as high-resource nations [3]. In Sweden, this issue is particularly relevant for Swedish-Somali women, as Somalia is the most common country of birth among Sub-Saharan Africans, with over 70,000 individuals living in Sweden [4].

Decreased fetal movements can be an indicator of fetal distress and is associated with adverse birth outcomes such as growth restriction [5]. The National Board of Health and Welfare in Sweden recommends that information about fetal movements is provided to all pregnant women within the antenatal care programme that the majority of women attend [6]. Approximately one in five women in Sweden seek hospital care for decreased fetal movements at some point during pregnancy [7]. However, findings from a previous study indicate that women born in Somalia seek care for decreased fetal movements less frequently than Swedish-born women [8]. Additionally, migrant women are at higher risk of inadequate antenatal care, including missed ultrasound screenings, delayed or lower attendance in the antenatal care programme, and missed postpartum care appointments [1, 9]. This is reflected in the overrepresentation of migrant women in preventable stillbirth cases, where timely and adequate care could have altered the outcomes [10]. It is possible that increased awareness of fetal movements among these women could improve the chances of identifying babies in distress and enable for timely obstetric interventions that could save lives.

Two Swedish studies from our research group have identified deficiencies in the information provided about fetal movements to migrant women in antenatal care [11, 12]. Women who do not receive information about fetal movements in their first language struggle to comprehend and retain it [11]. Further, midwives report that the antenatal care programme does not adequately support them in providing individualised information and care regarding fetal movements [12]. A previous randomised controlled trial (RCT) [13] conducted in Sweden between 2016 and 2018 found that women who were encouraged to become familiar with their unborn baby’s movement pattern using the Mindfetalness concept [14] increased their healthcare contacts for decreased fetal movements. That trial forms the foundation for the design of the intervention used in this study, except that here we focus solely on women born in Somalia.

With the aim to reduce maternal health inequities in Sweden, the National Board of Health and Welfare highlight the need for specific interventions targeting women at risk of adverse pregnancy and birth outcomes [15]. Recognising the existing disparities and the potential for targeted strategies to improve maternal and newborn health, this study aimed to evaluate the effect of a Mindfetalness-based intervention in increasing awareness of fetal movements and its impact on birth outcomes among Swedish-Somali migrant women.

## Methods

### Setting

In Sweden, healthcare is governed at a regional level through 21 regions that have the primary responsibility for health and medical care within their respective geographic area [16]. Maternity care is publicly funded, and pregnant women are offered care according to an antenatal care programme that recommends a minimum of nine care appointments to a midwife during pregnancy. The purpose of the program is to promote a healthy pregnancy and enable for early detection of pregnancy complications. The midwife serves as the primary care provider and manages regular appointments. If complications or increased risks are identified, additional appointments are added, and the midwife collaborates with other healthcare professionals such as physicians [15]. Sweden strives for equitable maternity healthcare through national guidelines and a centralised knowledge management system. However, regional adaptations of these guidelines occur [16], resulting in variations in the frequency of care appointments within the antenatal care programme and in diagnostic criteria for pregnancy-related complications, which have implications for how pregnancies are monitored and managed. In Sweden, individuals who do not speak Swedish are entitled to an interpreter free of charge when in contact with healthcare services [17].

In recent years, there has been the substantial increase in births starting with induction. From 2019 to 2020, the proportion of induced births rose from 19 to 25%. The largest increase occurred in gestational week 41, where the proportion of births starting with induction doubled, from 21% to 42% [18]. This increase can be attributed to new guidelines recommending that women should either be in labour or have given birth before reaching 42 weeks gestation [19]. Additionally, updated guidelines for managing complicated pregnancies often suggest inducing labour no later than the estimated due date [20]. Combined with the fact that in 2023, only 23% of all women had no identified risk factors at their initial maternity care registration [21], this likely contributes to the significant increase in labour inductions seen across Sweden.

### Design and sample

We conducted an intervention study with a non-randomised control group [22], encompassing Swedish-Somali migrant women with a singleton pregnancy, giving birth from gestational week 32 + 0 onwards between 1 June 2022 and 30 June 2024. Data were collected from the Swedish Pregnancy Register and the Swedish Neonatal Quality Register. The study was registered at ClinicalTrials.gov on 30 June 2022 (NCT05540639). Ethical approval was obtained from the Swedish Ethical Review Authority (approval reference No. 2021 − 00743, 2022-07005-02, and 2024-00494-02).

Midwifery clinics with ten or more women born in Somalia registered in 2019 were invited to participate in the intervention. Of the 40 clinics invited in November 2021, 26 chose to participate. Additionally, eight clinics from the same catchment area that wished to join were accepted, bringing the total number of clinics included in the intervention to 34, representing 11 different regions. The primary reasons given for declining participation were ongoing involvement in other clinical trials or strained workload that made the introduction of a new working method inappropriate. The control group consisted of women who gave birth during the same time as the intervention was implemented, but who were registered at midwifery clinics that had not been invited or had declined participation in the intervention. The control group included 287 midwifery clinics, located in 19 different regions.

### The mindfetalness intervention

The intervention aimed to improve the availability, accessibility, and women’s receptiveness to information about fetal movements, raise awareness of fetal movements as an indicator of the unborn baby’s wellbeing, and encourage women to become familiar with their baby’s movements pattern through the Mindfetalness concept [14]. Mindfetalness practice is recommended from gestational week 28 + 0 onwards and involves lying on the left side for 15 min daily during a period when the baby is active. During this time, the woman focuses on three aspects of fetal movements: strength, character, and frequency, without counting each movement.

Based on the findings from the original Mindfetalness trial [13], we hypothesised that a modified Mindfetalness-based intervention tailored specifically for women born in Somalia would enhance awareness of fetal movements, leading to an increase in healthcare contacts for decreased fetal movements and, ultimately, improved birth outcomes. The intervention design was adapted from the original trial by adding a video featuring a Somali woman, with the information narrated in Somali, to the Mindfetalness material. We also added educational components to strengthen the participating midwives’ knowledge of pregnancy-related risks specific to the target population. The components and intended mechanisms of the intervention are presented in a logic model (Supplementary Fig. 1).

**Fig. 1:**
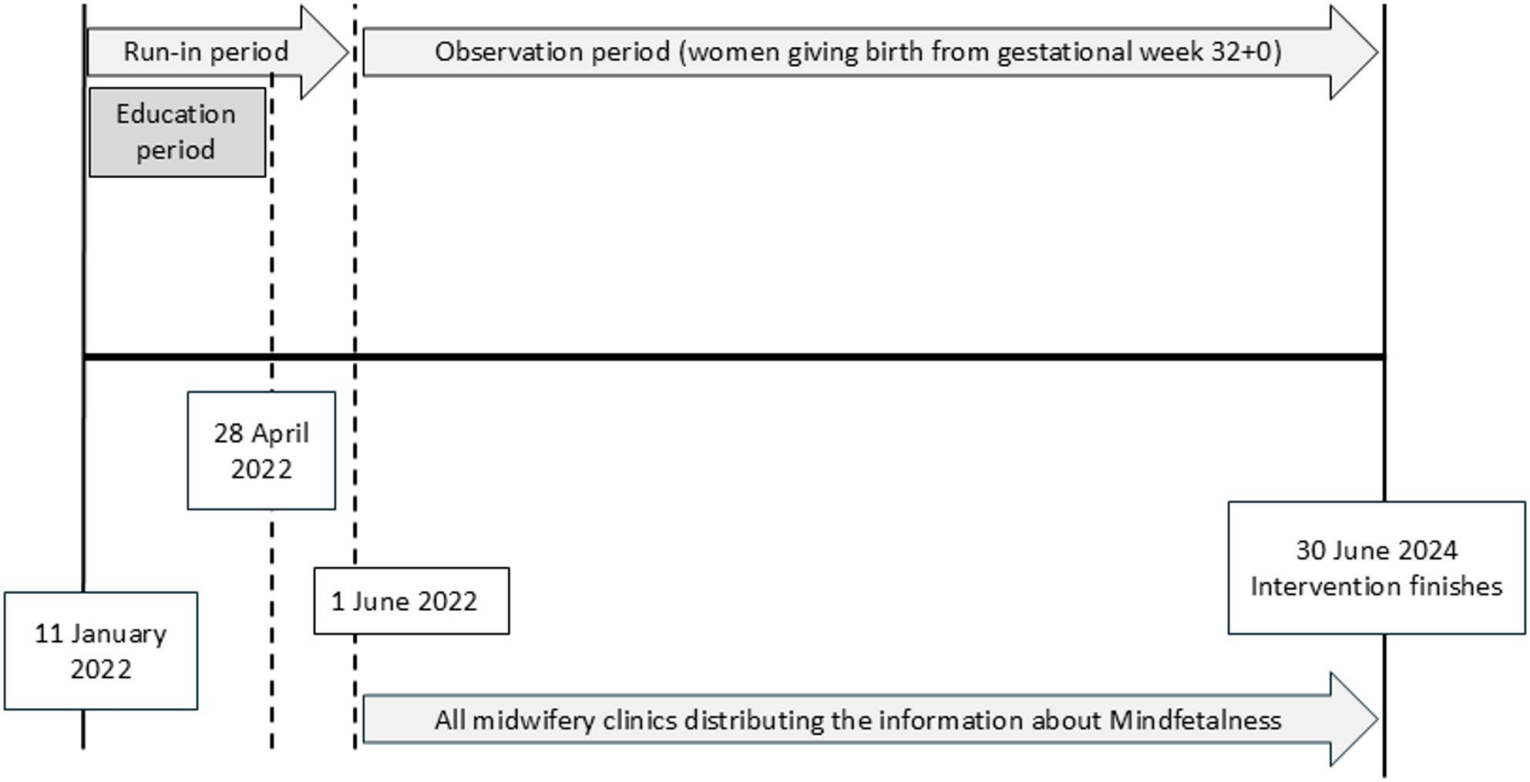
Trial flow chart

The implementation of the intervention began in January 2022, with a gradual roll-out across participating clinics (Fig. 1). The observation period started on 1 June 2022. Before that, all enrolled midwifery clinics received an introductory lecture on fetal movements, Mindfetalness, and pregnancy-related complications for Swedish-Somali migrant women. Throughout the spring of 2022, transmission from the Coronavirus disease 2019 (Covid-19) remained high, impacting healthcare routines as well as general recommendations for social encounters [23]. Consequently, some of these lectures were held digitally instead of in person.

The intervention proceeded in such way that the midwife handed out a leaflet about Mindfetalness to women who met the inclusion criteria in gestational week 24. The women were encouraged to start practicing Mindfetalness in gestational week 28 + 0 and continue until the baby was born. They were informed that practicing Mindfetalness was optional and entirely their decision. If the midwife first encountered the woman later in pregnancy, the leaflet was handed out then. The leaflet was available in Swedish and Somali. A short video presenting Mindfetalness was distributed in both languages on a website, which could be accessed using a QR-code on the back of the leaflet. During the intervention period, a monthly newsletter with information about the project and summaries of research findings within the field of fetal movements and migrant health were sent out by email to the midwives involved (a total of 19 letters). Additionally, findings from two studies exploring factors influencing awareness of decreased fetal movements [11, 12] were presented to each midwifery clinic through digital or physical lectures. Women in the control group had received routine information about fetal movement awareness according to regional guidelines, generally consisting of advising women to be observant of the baby’s movement pattern and to seek care if they experience decreased or altered movement pattern [6].

### Outcomes

Our initial intention was to evaluate women’s healthcare-seeking behaviour for decreased fetal movements using the Swedish classification of health procedures (KVÅ) code AM041 (*Examination due to decreased fetal movements*) [26]. According to the Swedish National Board of Health and Welfare, the proportion of women examined for decreased fetal movements is a key quality indicator for care related to stillbirth [6]. After personal contact between the last author (AAk) and healthcare professionals from different healthcare regions with knowledge about diagnostic coding, we unfortunately had to conclude that there were significant deficiencies in how this code was used across several regions, and therefore this outcome was excluded from the analysis.

The primary outcome measure was Apgar score below 10 at five minutes after birth, as this has been associated with increased neonatal mortality and morbidity when compared to an Apgar score of 10 at five minutes [24]. Secondary outcome measures included, in line with recommendations from the Swedish perinatal core outcome set [25], Apgar score below seven and four at five minutes after birth, low birth weight, neonatal intensive care unit (NICU) admission, death (fetal or within 27 days after birth), onset of labour, mode of birth, and gestational age at birth. Induction of labour were classified based on indication, separating fetal indication (e.g. suspicion of fetal distress or compromise) from induction due to prolonged pregnancy (e.g. pregnancies exceeding 40 + 0 weeks of gestation).

### Statistical analysis

The analysis included women who gave birth from gestational week 32 + 0 onwards from 1 June 2022 to 30 June 2024. The power calculation was based on unpublished findings from Akselsson et al. [13]. To detect a difference between the intervention and control groups in the primary outcome (Apgar score < 10 at 5 min after birth) we calculated that a sample size of 1,175 women in each group would provide 80% statistical power to detect a reduction from 16% to 12% (significant level set to 0.05, two tailed test).

The study sample consisted of 2,806 women, with 1,251 in the intervention group and 1,555 in the control group. Fetal indications for induction were identified using the following diagnostic codes from the international classification of diseases, 10th revision (ICD-10) [26]: signs of hypoxia (O363); known or suspected intrauterine growth retardation (O365); known or suspected specified problems of the fetus (O368); other specified (suspected) problems of the fetus (fetal heart arrhythmia) (O368B); known or suspected problems of the fetus (O368W); known or suspected problem of fetus, unspecified (O369), and oligohydramnios (O410). Induction due to prolonged pregnancy was determined based on the diagnostic code for labour induction (O61) in combination with the code for prolonged pregnancy (O49.0 or O.49.1).

To evaluate differences in the distribution of variables of interest between the two groups, we applied the Fisher’s exact test. As a measure of association, we calculated percentage ratios, which are presented as risk ratio (RR) or adjusted risk ratio (aRR). To account for potential confounding factors, we used a log-binomial regression model to adjust the RR and calculate 95% confidence intervals. The confounding factor considered in this analysis included educational level. Additionally, we stratified the sample based on parity. Statistical analyses were performed using the R statistical software (version 4.3.2).

## Results

Educational level differed between the intervention and control groups (p = < 0.001) (Table 1). There were more women in the intervention group with high school education (42.4% vs. 36.4%) and fewer with educational level shorter than nine years (12.6% vs. 15.4%). Occupation status also differed significantly (*p* = 0.001).

**Table 1:** Demographic characteristics among 1,251 women in the intervention group and 1,555 women in the control group

Demographic characteristics	Intervention group *N* = 1,251 *n* (%)	Control group *N* = 1,555 *n* (%)	*P*-value
Parity			0.555
Primiparous	273 (21.8)	355 (22.8)	
Multiparous	978 (78.2)	1200 (77.2)	
Maternal age			0.966
≤ 24 years	266 (21.3)	327 (21.0)	
25–34 years	639 (51.1)	802 (51.6)	
≥35 years	346 (27.7)	426 (27.4)	
Educational level			< 0.001
University	148 (11.8)	158 (10.2)	
High school	530 (42.4)	566 (36.4)	
Elementary school	243 (19.4)	305 (19.6)	
Shorter than 9 years	158 (12.6)	239 (15.4)	
Missing	172 (13.7)	287 (18.5)	
Occupation			0.001
Employed	553 (44.2)	685 (44.1)	
Student	257 (20.5)	309 (25.1)	
Parental leave	162 (12.9)	148 (9.5)	
Sickness absence	3 (0.2)	17 (1.1)	
Unemployed	138 (11.0)	162 (10.4)	
Other	75 (6.5)	106 (6.8)	
Missing	52 (4.5)	47 (3.0)	
Family situation^*^			
Cohabiting with the baby’s father	937 (75.2)	1143 (75.2)	1.00
Single parent	62 (5.0)	100 (7.0)	0.043
Other family situation	226 (18.3)	275 (19.0)	0.656
Body mass index			0.906
Underweight (≤ 18.4 kg/m²)	30 (2.4)	39 (2.5)	
Normal (18.5–24.9 kg/m²)	345 (27.6)	448 (28.8)	
Overweight (25.0–29.9 kg/m²)	442 (35.3)	539 (34.7)	
Obesity (≥ 30.0 kg/m²)	409 (32.7)	493 (31.7)	
Missing	25 (2.0)	36 (2.7)	
Tobacco/snuff use at registration at the midwifery clinic^*^	23 (1.9)	30 (2.0)	0.958
Previous stillbirth^*^	33 (3.4)	43 (3.6)	0.883
Medical history^*^			
Diabetes mellitus	16 (1.3)	44 (3.0)	0.005
Coronary heart disease	16 (1.3)	13 (0.9)	0.362
Chronic hypertension	7 (0.6)	13 (0.9)	0.480
Thrombosis	8 (0.7)	9 (0.6)	1.00
Endocrine disease	72 (5.9)	101 (6.9)	0.346
Gynecologic disease	430 (35.2)	352 (23.5)	< 0.001
Chronic kidney disease	1 (0.1)	3 (0.2)	0.754
Repeated urinary tract infections	64 (5.2)	56 (3.8)	0.085
Lung disease/asthma	61 (4.9)	84 (5.6)	0.476
Epilepsy	8 (0.6)	5 (0.3)	0.369
Jaundice	14 (1.1)	13 (0.9)	0.604
Mental illness	47 (3.8)	53 (3.6)	0.771
Medical and/or psychologic treatment for mental illness before and/or during pregnancy	12 (1.0)	30 (2.1)	0.058
Pregnancy related complications^*^			
Gestational diabetes	144 (12.3)	173 (11.9)	0.752
Preeclampsia	52 (4.2)	49 (3.2)	0.187
Gestational hypertension	28 (2.2)	52 (3.3)	0.102
*Missing values not included in the analysis			

Fewer women in the intervention group were students (20.5% vs. 25.1%), absence due to sickness was less common (0.2% vs. 1.1%), and more women were on parental leave (12.9% vs. 9.5%). There were significantly less women in the intervention group who were single parents (5.0% vs. 7.0%, *p* = 0.043). Regarding medical history and pregnancy related complications, there were more women in the intervention group with gynecological disease (e.g. fibroids, endometrioses, female genital mutilation) (35.2% vs. 23.5%). In both groups, there was a high prevalence of previous stillbirth among multiparous women, 3.4% in the intervention group and 3.6% in the control group. Further, two thirds were overweight or obese across both groups (68.0% vs. 66.4%), and almost one third had a prolonged or post-term birth (32.9% vs. 30.4%).

We found that more women in the intervention group had spontaneous vaginal birth with an aRR of 1.06 (CI 1.01–1.11) (Table 2). We found no statistically significant difference between the groups in any other birth outcomes. Stillbirth averaged 3.2 per thousand in both groups (0.4% vs. 0.3%).

**Table 2:** Birth outcomes among 1,251 women in the intervention group and 1,555 women in the control group

Outcome variables	Intervention group *N* = 1,251 *n* (%)	Control group *N* = 1,555 *n* (%)	aRR^*^ (95% CI)	*P*-value
Spontaneous onset of labour	687 (54.9)	882 (56.7)	0.96 (0.90–1.04)	0.339
Labour induction all causes	478 (38.2)	554 (35.6)	1.09 (0.98–1.21)	0.096
Labour induction fetal indication	107 (8.6)	114 (7.3)	1.03 (0.80–1.32)	0.843
Labour induction prolonged pregnancy	218 (17.4)	242 (15.6)	1.17 (0.97–1.40)	0.095
Spontaneous vaginal birth	953 (76.2)	1139 (73.2)	1.06 (1.01–1.11)	0.022
Instrumental vaginal birth	46 (3.7)	69 (4.4)	0.73 (0.49–1.08)	0.117
Elective cesarean section	90 (7.2)	122 (7.8)	0.88 (0.66–1.16)	0.352
Emergency cesarean section	161 (12.9)	221 (14.2)	0.91 (0.74–1.12)	0.385
Birth gestation < 37 + 0	43 (3.4)	42 (2.7)	1.30 (0.82–2.05)	0.264
Birth gestation 41 + 0–41 + 6	380 (30.4)	437 (28.1)	1.07 (0.94–1.21)	0.318
Birth gestation > 41 + 6	31 (2.5)	35 (2.3)	1.27 (0.75–2.17)	0.369
Birth weight < 2 SD	73 (5.8)	83 (5.3)	1.10 (0.78–1.54)	0.595
Birth weight < 10th centile	214 (17.1)	252 (16.2)	1.04 (0.86–1.24)	0.701
Apgar score < 10 at 5 min after birth	229 (18.3)	285 (18.3)	1.02 (0.86–1.21)	0.840
Apgar score < 7 at 5 min after birth	36 (2.9)	38 (2.4)	1.11 (0.67–1.84)	0.682
Apgar score < 4 at 5 min after birth	7 (0.6)	11 (0.7)	0.99 (0.32–2.97)	0.982
Admitted to neonatal intensive care unit	131 (10.5)	157 (10.1)	1.07 (0.84–1.36)	0.605
Neonatal mortality^**^	1 (0.1)	3 (0.2)	0.41 (0.02–3.23)	0.417
Stillbirth^**^	5 (0.4)	4 (0.3)	1.55 (0.41–6.27)	0.509
*Adjusted for educational level **Unadjusted data				

When stratifying women based on their reproductive history, for primiparous women, no statistically significant differences in birth or neonatal outcomes were found between the intervention and control groups. For multiparous women, birth outcomes followed the same pattern as the main analysis with a statistically significant increase in spontaneous vaginal births in the intervention group (aRR 1.05, CI 1.01–1.10).

## Discussion

### Main findings

In this non-randomised intervention study conducted in Sweden, encouraging women to monitor fetal movements by practicing Mindfetalness, we found a statistically significant increase in spontaneous vaginal births in the intervention group. We found no statistically significant differences in Apgar score at 5 min after birth or any other birth outcomes between the intervention and control groups. Two thirds of women were overweight or obese, and almost one third had a prolonged or post-term birth.

### Interpretation

We found that women who participated in the Mindfetalness-based intervention demonstrated higher rates of spontaneous vaginal births compared to women in the control group who received routine information about fetal movements. This finding aligns with the lower rates of caesarean sections found in the original Mindfetalness trial [13]. This discrepancy between the intervention and control groups can be due to regional variations in the incidence of spontaneous vaginal birth in Sweden, which in 2023 ranged from 67% to 85% across different hospitals [21]. It could, however, suggest that practicing Mindfetalness can have positive effects on the birth process. By reducing stress and anxiety while promoting relaxation [27], Mindfetalness practice may help prepare women for childbirth. Further research is needed to understand what mechanisms of Mindfetalness supports the birth process, and how midwives may integrate these mechanisms into antenatal care when providing information about fetal movements.

Like the original Mindfetalness trial [13], we found no improvements in Apgar score at five minutes after birth, or in any other neonatal outcomes among women who received targeted information about fetal movements and Mindfetalness. This may be attributed to an already high quality of standard care associated with decreased fetal movement in the Swedish maternity healthcare setting. Maternity healthcare in Sweden is well-organised, with access to standardised care programmes, educated midwives, and advanced neonatal care [28]. Fetal movement information is also well integrated in the antenatal care programme. This robustness of the healthcare system is supported by the relatively low proportion of stillbirths observed in both groups (0.4% in the intervention group and 0.3% in the control group). These rates are notably lower than the 1.1% observed among Somali-born women in the control group of the original Mindfetalness trial [29]. It is possible that midwives working in the midwifery clinics, despite not participating in the Mindfetalness-based intervention, through their knowledge and dedication to provide equal care, successfully managed to compensate for the barriers migrant women face when accessing information and care related to fetal movement awareness [12]. Implementing the Mindfetalness intervention in a high-burden context with substandard care would potentially have been more effective, demonstrating more positive effects on birth outcomes.

Compared to national averages, the women participating in our intervention had higher rates of overweight and obesity [21], which is associated with pregnancy related complication such as low Apgar score, fetal growth restriction, infant mortality, pre-term birth, gestational diabetes, pre-eclampsia, and prolonged and post-term birth [30, 31]. In our study population, almost one third of women had prolonged or post-term birth, which is significantly higher than the national average of 20% [21]. There was also a high prevalence of previous stillbirth across both groups, which is a risk factor for recurrent stillbirth, especially among Black women [32]. The educational level in our study population was, compared to national averages, low with only 10–11% of women having a university education [21]. Education is a measure of social positioning, which is an important determinant of health and health resources [33]. This elevated baseline risk is probably reflected in the high proportion of labour inductions observed: 38.2% in the intervention group and 35.6% in the control group, which can be compared to a national average of 29% in 2023 [21]. More risk factors may have required more medical interventions, potentially diminishing the effect of increased fetal movement awareness. While our intervention targeted one aspect of fetal well-being, it may not have been sufficient to overcome the many other factors and aspects influencing newborn health throughout pregnancy and childbirth. This highlights the need to consider baseline risk factors when designing and evaluating health interventions.

It is possible that our intervention did not adequately address the specific needs of this population, particularly in overcoming barriers in accessing information and healthcare services. The intervention targeted pregnant women and at midwives working in midwifery clinics, but did not involve healthcare professionals working in the hospitals where women seek care for decreased fetal movements. Previous research has identified that migrant women in Sweden face several barriers accessing hospital care related to decreased fetal movements [11, 12]. It is possible that engaging healthcare professionals across the entire care continuum would have improved the intervention’s effectiveness. Engaging stakeholders in the process of designing the intervention material and components could have resulted in a more tailored intervention, improving its acceptability among the target population, possibly enhancing its uptake and overall effectiveness [34].

The intervention’s multiple interacting components, including human factors, clinical guidelines, behavioural change, and contextual factors, presented challenges in both implementation and evaluation [35]. Further, the Covid-19 pandemic brought on increased workload [36] that may have affected the delivery of the intervention components. Evaluations of the MAMAACT intervention [37], which aimed to improve birth outcomes for migrant women in Denmark, found that organisational barriers including time pressure, lack of flexibility, and poor interpreter services influenced the delivery of the intervention negatively [38]. Despite finding the MAMAACT material useful, women’s response to signs of complication was also affected by lack of trust in healthcare providers, lack of social network and domestic responsibilities [39]. To fully understand why this intervention did not lead to significant improvements in birth outcomes, a process evaluation of contextual factors, implementation process, and mechanisms of impact from the perspective of the participants is needed [35]. Such evaluation could also guide future adaptions to the intervention that could improve its effectiveness and transferability to other settings [40].

### Methodological considerations

The design used for this study enabled a comparison of outcome measures despite the relatively small sample size. The use of quality registers with high coverage [41, 42] enabled inclusion of women from different parts of Sweden. The absence of randomisation limits the ability to control for confounding variables that may have influenced the outcomes [43]. This limitation potentially introduces bias, making it challenging to establish a clear causal relationship between the intervention and the observed effects. To mitigate this risk, we adjusted for educational level as a potential confounding factor in the statistical analysis. Although the intervention and control groups were comparable on most measured characteristics, we cannot, however, rule out the influence of unmeasured confounders, including differences in site characteristics and the varying density of Somali-born women across sites, which may have affected the results. By using a control group consisting of women giving birth during the same time period as the intervention group, we reduced the risk of potential confounding due to changes in practice, policies, or other factors that may occur over time [22]. We could, however, not control for regional differences in guidelines and protocols. We cannot rule out the possibility that both women and midwives in the control group accessed information about Mindfetalness, as it is publicly available through an open website. This presents a risk of contamination, potentially leading to unintended effects in the control group, possibly diluting the observed differences between the groups.

There is a potential for selection bias [44], as only 26 out of 40 invited midwifery clinics participated. The participating clinics may not be representative of all midwifery clinics, potentially limiting the generalisability of the findings. They may have been more motivated or better resourced, possibly leading to an overestimation of the intervention’s effectiveness. Additional clinics that wished to join were accepted, which may further have biased the sample towards more motivated clinics.

We hypothesised that our intervention would positively affect birth outcomes by increasing women’s awareness of decreased fetal movements, enabling early identification of babies in distress and timely interventions to prevent adverse birth outcomes. Unfortunately, due to deficiencies in the application of the KVÅ code AM041 across several regions, we were unable to evaluate this aspect within the scope of this study. Information on this outcome would have contributed to valuable knowledge in understanding the mechanisms of the intervention and in interpreting the study findings. It is noteworthy that the majority of regions in Sweden do not adhere to the recommendations for diagnostic coding developed by the National Board of Health and Welfare [6].

The lack of information on how many women received information about Mindfetalness and chose to practice it as intended is a limitation that could lead to over- or underestimation of the intervention’s effectiveness. Akselsson et al. [45] reported that 75% of 104 Swedish-speaking women who received Mindfetalness information practiced the method daily, with higher compliance among primiparous (88%) than multiparous women (64%). However, the study’s homogenous sample—mostly Swedish-born and high educated women—differs with the demographic characteristics of our intervention group, complicating the estimation of how many women in our population chose to practice Mindfetalness. Without accurate information regarding frequency of practice, there is a risk of misclassifying women’s exposure to the concept, making it difficult to assess any dose-response relationship.

## Conclusions

In this modified Mindfetalness-based intervention, which included 2,806 Swedish-Somali migrant women giving birth in Sweden, a higher proportion of women in the intervention group had a spontaneous vaginal birth compared to the control group. The intervention did not significantly impact any other birth outcomes. The implementation and evaluation of complex interventions in a high-quality healthcare setting, such as Swedish maternity care with its standardised care guidelines and protocols, present challenges. When designing health interventions to improve birth outcomes, it is important to account for baseline risk factors to ensure their effectiveness and alignment with the specific needs of the target population. Further research involving key stakeholders representing Swedish-Somali women and midwives working in antenatal care is essential to understand and evaluate the intervention’s broader implications. This research should also focus on identifying necessary adaptations to ensure its successful implementation within this specific population and healthcare setting.

## Supplementary Information

Supplementary Material 1.

## Data Availability

Data are not publicly available. Anonymised data are available after ethical approval by the Swedish Ethical Review Authority and after request to the Swedish Pregnancy Register and the Swedish Neonatal Quality Register.
