# Supplementary figures and images for: The effect of fetal movement awareness on birth outcomes among Somali migrant women – findings from a non-randomised intervention study in Sweden
Source: BMC Pregnancy Childbirth. 2026 Jan 28;26:189. doi: 10.1186/s12884-026-08707-z (PMC12924534; doi:10.1186/s12884-026-08707-z)

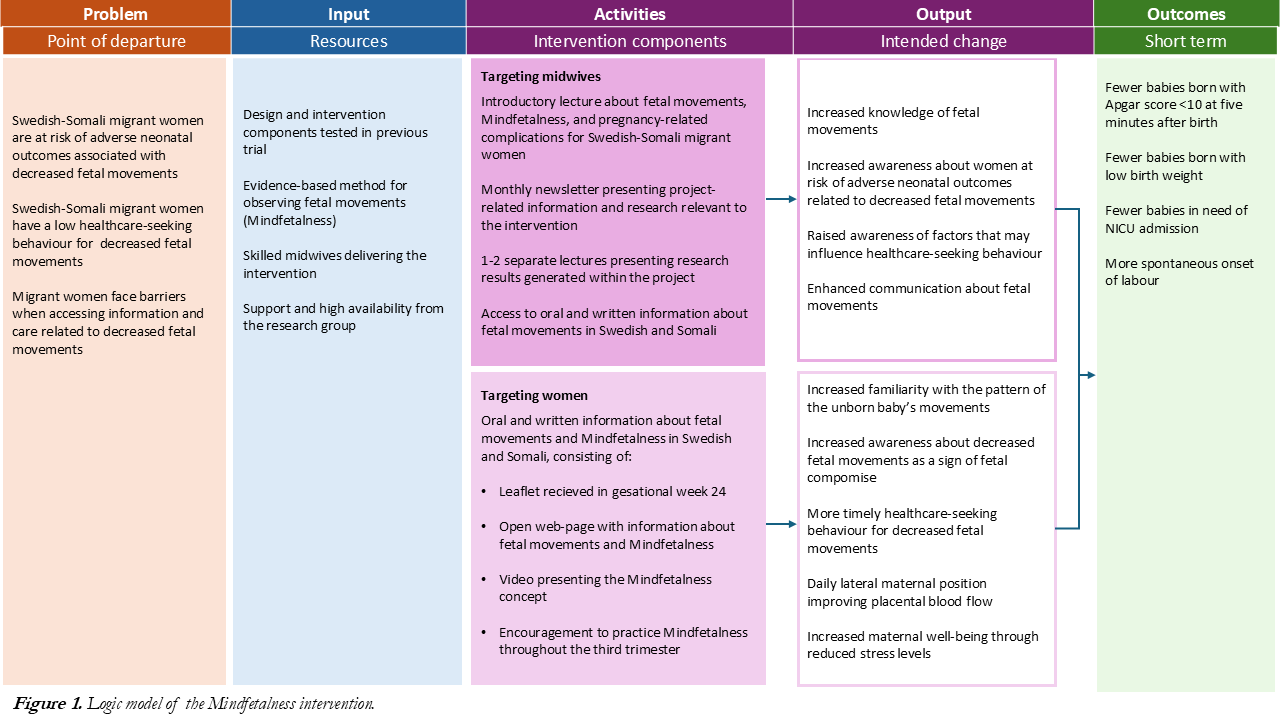

Supplement: Supplementary file 1 — Supplementary Material 1. [file 12884_2026_8707_MOESM1_ESM.tif]
